# Exploring the causal role of gut microbiota in giant cell arteritis: a Mendelian randomization analysis with mediator insights

**DOI:** 10.3389/fimmu.2023.1280249

**Published:** 2024-01-04

**Authors:** Menglin Wu, Zhixiong Liao, Kaidong Zeng, Qiaohui Jiang

**Affiliations:** ^1^ Department of Cardiology, Zhangjiajie People’s Hospital, Zhangjiajie, China; ^2^ Department of Cardiology, The Second People’s Hospital of Neijiang, Neijiang, China

**Keywords:** 25 hydroxyvitamin D, giant cell arteritis, gut microbiota, immune cell, mediation analysis, Mendelian randomization

## Abstract

**Background:**

Giant Cell Arteritis (GCA) is a complex autoimmune condition. With growing interest in the role of gut microbiota in autoimmune diseases, this research aimed to explore the potential causal relationship between gut microbiota and GCA, and the mediating effects of specific intermediaries.

**Methods:**

Using a bidirectional two-sample Mendelian randomization (MR) design, we investigated associations between 191 microbial taxa and GCA. A two-step MR technique discerned the significant mediators on this relationship, followed by Multivariable MR analyses to quantify the direct influence of gut microbiota on GCA and mediation effect proportion, adjusting for these mediators.

**Results:**

Nine taxa displayed significant associations with GCA. Among them, families like Bacteroidales and Clostridiaceae1 had Odds Ratios (OR) of 1.48 (p=0.043) and 0.52 (p=5.51e-3), respectively. Genera like Clostridium sensu stricto1 and Desulfovibrio showed ORs of 0.48 (p=5.39e-4) and 1.48 (p=0.037), respectively. Mediation analyses identified 25 hydroxyvitamin D level (mediation effect of 19.95%), CD14+ CD16- monocyte counts (mediation effect of 27.40%), and CD4+ T cell counts (mediation effect of 28.51%) as significant intermediaries.

**Conclusion:**

Our findings provide invaluable insights into the complex interplay between specific gut microbiota taxa and GCA. By highlighting the central role of gut microbiota in influencing GCA risk and long-term recurrence, and their interactions with vital immune mediators, this research paves the way for potential therapeutic interventions in GCA management.

## Introduction

Giant cell arteritis (GCA), the most prevalent form of vasculitis in Western countries (1), most patients experience symptom relief within days of starting corticosteroid therapy. However, relapses can occur, especially during the tapering of corticosteroids (2). The most feared complication is irreversible vision loss, which can occur suddenly if the ophthalmic artery or its branches become involved. Other neurological complications include stroke and transient ischemic attacks. Additionally, GCA can lead to aortic aneurysms, particularly of the thoracic aorta, and aortic dissections. Patients may also experience systemic symptoms such as fatigue, weight loss, and fever, which can significantly impact their quality of life (3)

The underlying GCA remains elusive, but the disease is fundamentally characterized by inflammation within the medium to large arteries branching from the aortic arch. The pathogenesis of GCA is believed to involve an aberrant reaction of the immune system to damage within the vascular endothelium, exhibiting a predominantly Th-1 mediated immune response with a notable increase in IFN-Gamma production. Recent insights have also indicated a role for Th-17 responses, which appear to be more susceptible to glucocorticoid therapy (4). The disease process initiates when an initial endothelial insult, which could be due to physical injury, infection, a reaction to a medication, or an autoimmune response, activates dendritic cells in the arterial adventitia. These cells, in turn, secrete chemokines that recruit CD4+ helper T cells and macrophages to the site. Further, the release of IL-6 and IL-18 by these dendritic cells triggers T-cell activation and subsequent release of IFN-gamma, fueling inflammation, encouraging macrophage activity, and leading to the formation of granulomas. Macrophages respond by producing matrix metalloproteinases and reactive oxygen species, inflicting further endothelial damage and disrupting the internal elastic lamina. Additionally, they produce nitric oxide, which, in conjunction with other macrophages, contributes to the formation of the characteristic multinucleated giant cells. These activated macrophages not only perpetuate local vascular inflammation but also have systemic effects by releasing pro-inflammatory cytokines such as IL-1 and IL-6 (5, 6).

The etiology of GCA remains incompletely understood, but it is believed to involve a combination of genetic predisposition and environmental triggers. Recently gut microbiota, a complex community of microorganisms residing in the human gastrointestinal tract, has been increasingly recognized for its pivotal role in health and disease (7). It not only aids in digestion and metabolism but also plays a crucial role in shaping the host’s immune system. Recent studies have highlighted the potential role of the gut microbiota in modulating immune responses and influencing the development of various autoimmune diseases, including GCA (8, 9). Dysbiosis have been linked to a plethora of diseases ranging from metabolic disorders to autoimmune conditions (10). Futhermore, recently study find Vitamin D involved in these processes might serve as potential mediators in the relationship between the gut microbiota and GCA (11).

Mendelian randomization (MR) is a method that uses genetic variants as instrumental variables to infer causality between an exposure and an outcome. By leveraging this approach, we aimed to investigate the causal relationship between the gut microbiota composition and the risk of GCA (12). Considering the intricate interplay between the metabolism, immune responses, and the gut microbiota, we further selected 25 hydroxyvitamin D level and 731 immune cell traits to explore their potential mediation effects in this association.

By evaluating these mediation effects, we can gain insights into the mechanistic pathways through which the gut microbiota might influence the risk of GCA, and recurrence risk of GCA in a long term, paving the way for potential therapeutic interventions.

## Methods

### Study design

**Figure 1:**
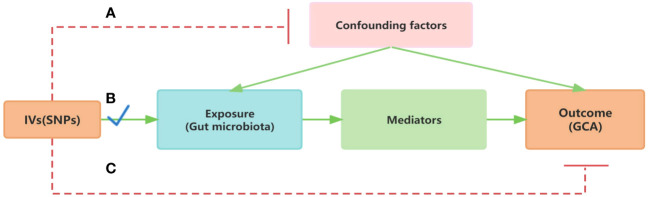
Mendelian Randomization assumption plot. **(A)** IVs are independent of confounders; **(B)** IVs have a strong association between IVs and the exposure; **(C)** IVs affect the outcome only through the exposure.

**Figure 2:**
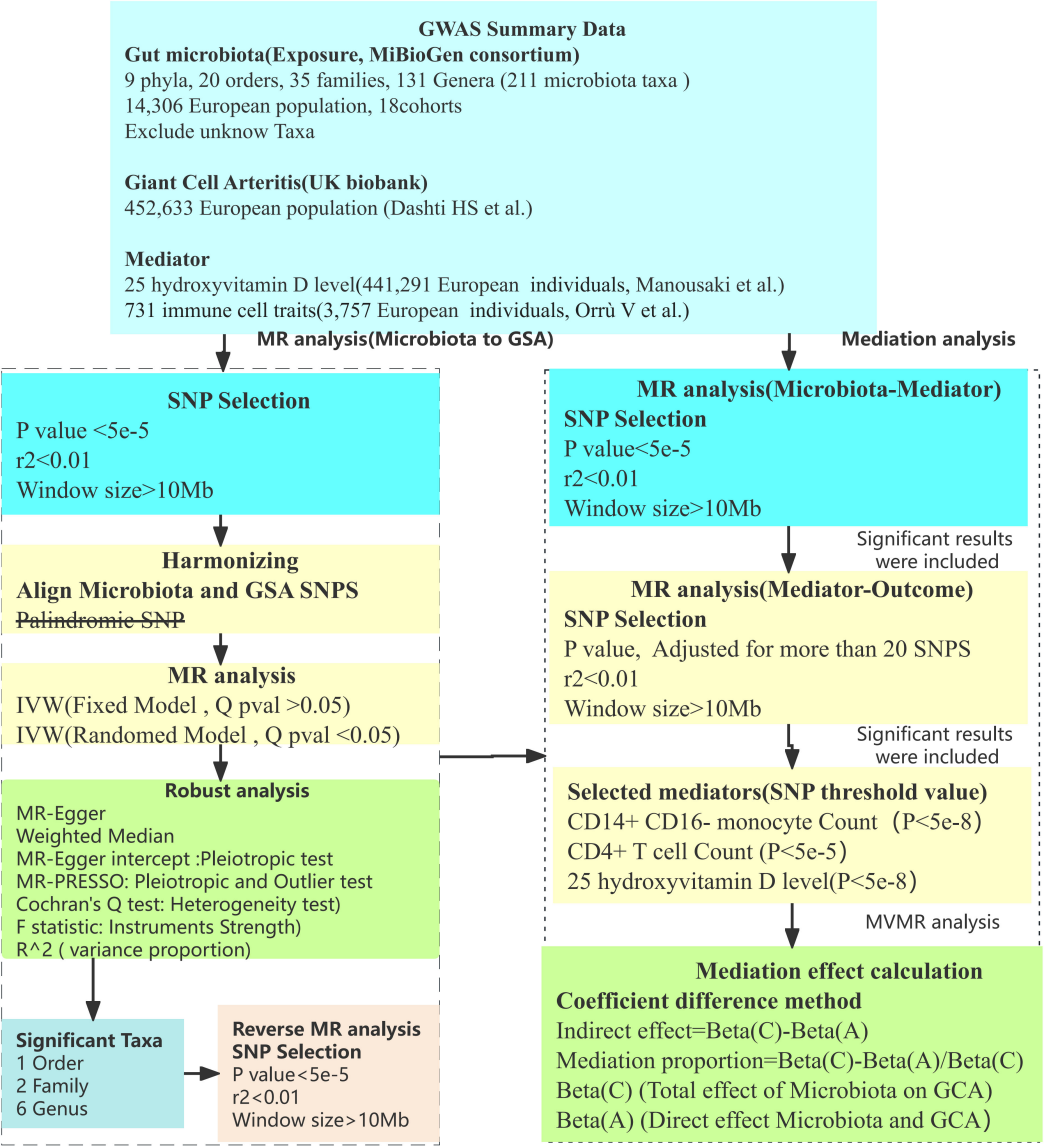
Flow chart. Outlines the methodology used to investigate the link between gut microbiota and GCA via GWAS data. SNP selection criteria were applied before harmonization for MR analysis to determine the causal relationships and identify significant microbial taxa. Mediation analysis further quantified the potential influence of immune cell traits, 25 hydroxyvitamin D level on the microbiota-GCA association.

Utilizing a two-sample MR methodology, we first probed the causal associations between gut microbiota and GCA. This method(**Figure 1**) uses genetic variants, particularly single nucleotide polymorphisms (SNPs), as instrumental variables (IVs). The efficacy of the MR analysis hinges on three central assumptions(**Figure 1**): a) IVs are independent of confounders; b) There’s a strong association between IVs and the exposure; c) IVs affect the outcome only through the exposure. To ensure the robustness and directionality of our findings, robust analysis and reverse MR analysis was also performed. Given the intricate interplay between immune responses and gut microbiota, we further selected 25 hydroxyvitamin D level and 731 immune cell traits as potential mediators. A two-step (network) MR approach was implemented to identify significant mediators among these (13). Subsequently, using Multivariable MR (MVMR), we estimated the direct effect of gut microbiota on GCA, adjusting for the significant mediators (14). This allowed us to calculate the proportion of the effect of gut microbiota on GCA that is mediated through these 25 hydroxyvitamin D and immune mediators. The flow chart illustrating our study design and analytical steps is depicted in **Figure 2**. Through this rigorous design, our study aims to provide deeper insights into the role of gut microbiota in GCA pathogenesis, highlighting potential therapeutic targets and pathways of GCA.

### Data sources

Our research made use of multiple datasets to investigate the intricate relationships between gut microbiota, GCA, 25 hydroxyvitamin D, and immune cell traits with various health outcomes. Gut microbiota (15) from the comprehensive MiBioGen consortium, which analyzed genome-wide genotypes in conjunction with 16S fecal microbiome data spanning 18,340 individuals from 24 distinct cohorts. Significantly, 14,306 of these individuals, spread across 18 cohorts, are of European ancestry. For insights into GCA, we harnessed data from the UK Biobank, encompassing 278 cases against a backdrop of 456,070 controls. 25 hydroxyvitamin D level was obtain from Manousaki et al. (16), including 441,291 samples. Diving deeper into the genetic nuances of immune functionality, we incorporated a dataset from Orrù V et al. that provided a breakdown of 731 immune cell traits based on an analysis of over 3,000 individuals (17). Ensuring consistency, all participants in our study hail from European descent, with extensive details and direct link captured in **Supplementary Table 1.**

### SNP selection

SNPs that displayed a genome-wide significant association (p < 5 × 10^−8) from the GWAS summary data for each exposure were chosen as potential IVs. Owing to a limited number of available IVs, we modified the significance threshold to p < 5 × 10^−5. Specifically, the thresholds were set at: P<5e-5 for both gut microbiota and GCA. For the analysis involving 25 hydroxyvitamin D level and the 731 immune cell traits, we tailored the significance threshold based on a criteria requiring more than 20 SNPs. The mediators identified in our study were 25 hydroxyvitamin D level (P<5e-8), CD14+ CD16- monocyte (P<5e-8), and CD4+ T cell Count (P<5e-5).

Subsequent steps in our methodology encompassed linkage disequilibrium clumping using the criteria of r^2 < 0.01 within a window size of >10,000 kb (18), harmonization of the exposure and outcome datasets, and purging of palindromic SNPs with allele frequencies approaching 0.5. To guarantee the robustness of our selected genetic instruments, we computed the F statistic with the formula (19): F = R^2 × [(N – 1 − k)/k] × (1 − R^2). Here, R^2 is the aggregate variance attributed to the chosen SNPs, N stands for the sample size, and k indicates the number of SNPs in our analysis. An F-statistic above 10 is indicative of a solid instrument, addressing potential weak instrument bias in our two-sample model. Comprehensive details on the chosen SNPs are documented in **Supplementary Table 2**.

### Statistical analysis

We conducted bidirectional two-sample MR analyses to elucidate the relationships between gut microbiota and GCA, and their potential mediation by 25 hydroxyvitamin D and immune cell traits. Central to our methodology was the inverse variance-weighted (IVW) method, recognized for its robustness in MR studies (20). To reinforce the reliability of our conclusions, supplementary analyses, including the weighted median(this approach assumes that at least half of the SNPs are free of pleiotropy) (21) and MR-Egger regression techniques(this approach no limited bu the pleiotropy proportion of SNP) (22), were executed. The potential presence of directional pleiotropy was scrutinized via the intercept value of the MR-Egger regression. Additionally, Mendelian randomization pleiotropy residual sum and outlier(MR PRESSO) was employed to inspect outliers (23).To gauge heterogeneity among our data sets, we utilized Cochran’s Q test (24). In cases of evident heterogeneity, we transitioned to a random-effects IVW as our primary analysis approach. The results of the main MR analysis (*P* < 0.05) was considered as significance.

## Results

### MR analysis of gut microbiota’s effect on GCA

**Figure 3:**
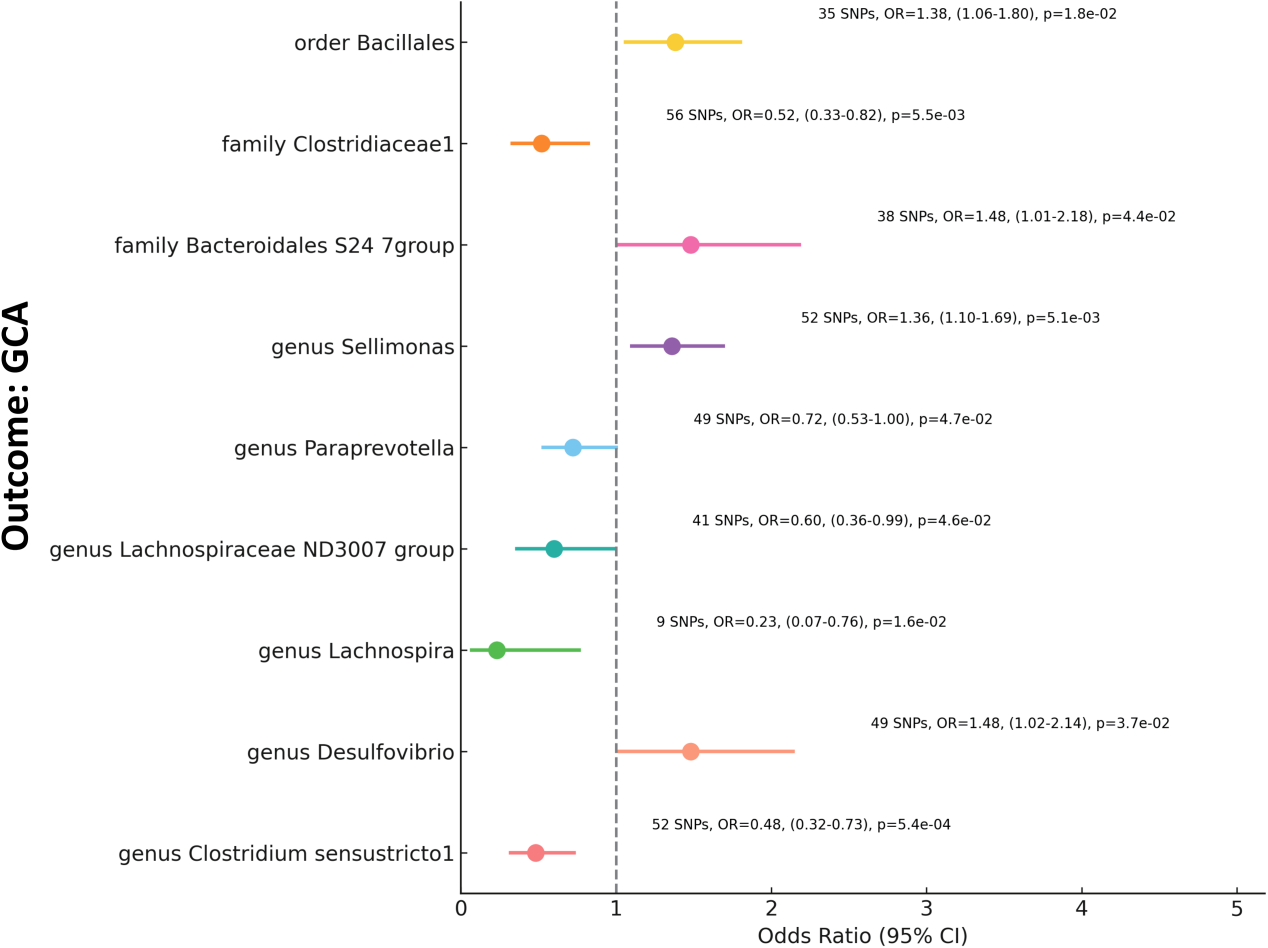
Mendelian Randomization Analysis of Gut Microbiota and GCA This forest plot visualizes the relationship between specific gut microbial taxa (Exposure) and GCA (Outcome). Each line represents a distinct microbial taxon-GCA pair. The plot markers signify the OR value, while the horizontal lines provide the 95% confidence intervals. Annotations on the right side detail the number of SNPs used, the Beta value, the confidence interval range, and the p-value for each relationship. A vertical dashed line at OR=1 serves as a reference point, indicating no effect.

In our MR analysis examining the relationship between gut microbiota and GCA, we assessed 211 microbial taxa. Post removal of 20 unidentified taxa, the remaining 191 were scrutinized, yielding significant associations for nine specific taxa (**Figure 3**, **Supplementary Table 3**). Notably, families such as Bacteroidales S24-7group and Clostridiaceae1 demonstrated Odds Ratios (OR) of 1.48 (p=0.043) and 0.52 (p=5.51e-3), respectively. Among the genera, Clostridium sensustricto1 showed an OR of 0.48 (p=5.39e-4), Desulfovibrio an OR of 1.48 (p=0.037), Lachnospiraceae ND3007 group an OR of 0.6 (p=0.046), Lachnospira an OR of 0.23 (p=1.59e-2), Paraprevotella an OR of 0.72 (p=0.047), and Sellimonas an OR of 1.36 (p=5.10e-3). Furthermore, from the order category, Bacillales exhibited an OR of 1.38 (p=0.018). These findings underline the intricate interplay between specific constituents of the gut microbiota and GCA, emphasizing the potential influence of gut microbiome diversification on autoimmune conditions.

**Figure 4:**
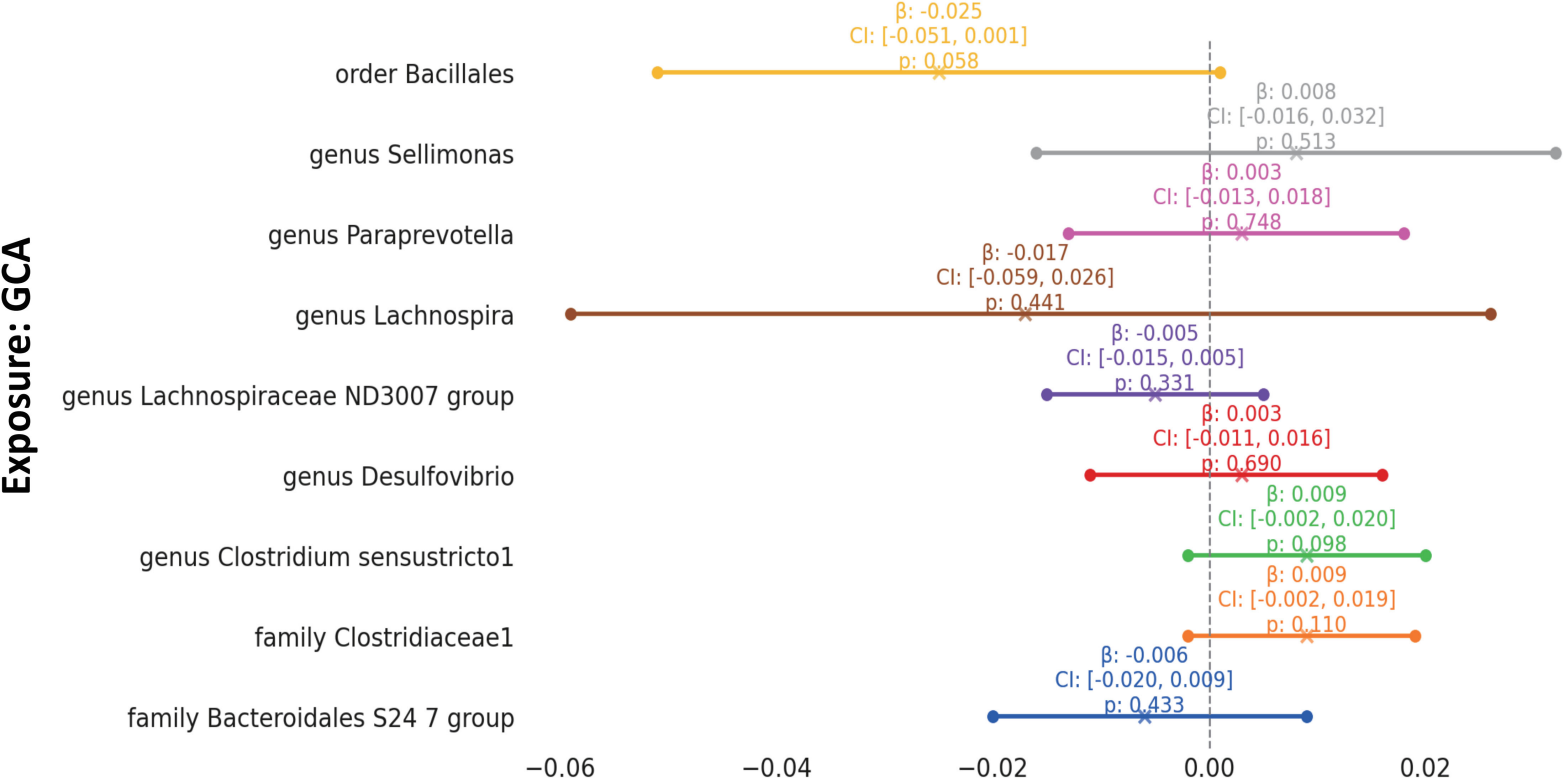
Mendelian Randomization Analysis of GCA on Gut Microbiota This forest plot visualizes the reverse relationship between specific gut microbial taxa (Exposure) and GCA (Outcome). Each line represents a distinct GCA-microbial taxon pair. The plot markers signify the beta value, while the horizontal lines provide the 95% confidence intervals. A vertical dashed line at Beta=0 serves as a reference point, indicating no effect.

To enhance the rigor of our analysis focused on the interplay between specific gut microbiota taxa and GCA, supplementary analyses were carried out, as detailed in **Supplementary Table 3**. Significantly, the results from the Weighted Median (WM) and MR-Egger methodologies were largely in alignment with the findings from the Inverse Variance Weighted (IVW) method, providing additional weight to our conclusions. The R^2 values indicated that these selected SNP explain between 1.1% and 7.0% of the variance in taxa. Impressively, all of the F statistics exceeded 10, underlining the robustness of our instrumental variables. Through the use of MR-PRESSO (**Supplementary Table 4**), we detected no outliers, further cementing the consistency and dependability of the associations we delineated. The intercept tests, as illustrated in **Supplementary Table 3**, revealed no significant pleiotropy. This provides confidence that the genetic variants we selected have effects on GCA predominantly through the gut microbiota taxa we investigated. Intriguingly, in the reverse MR analysis, no significant influence was found of GCA on the gut microbiota composition (**Figure 4**, **Supplementary Table 5**).

### Mediation analysis of gut microbiota on GCA

**Figure 5:**
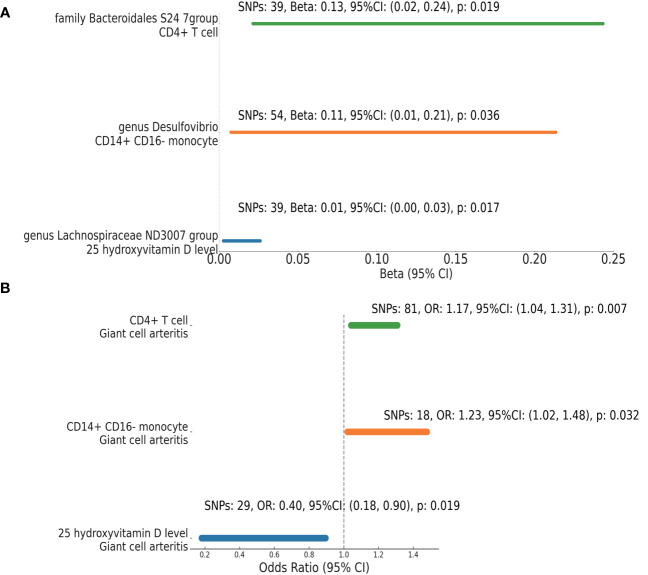
Mendelian Randomization Analysis: Microbiota’s Effect on Mediators and Mediators’ on GCA. This figure visualizes the results from a MR analysis investigating **(A)** the effect between microbial taxa and specific mediators; **(B)** the effect between mediators and GCA. Each line represents a different microbial taxa-mediator or mediator-GCA pair. The endpoints of the lines indicate the 95% confidence intervals for the effect size (Beta or OR), while the numbers alongside provide detailed statistics including the number of SNPs used, the Beta, OR value, its 95% confidence interval, and the corresponding p-value. Notably, the effect sizes and their confidence intervals are presented on the x-axis.

In our investigation to elucidate the mediating effects of gut microbiota on GCA via 25 hydroxyvitamin D and immune traits, we employed a two-step MR approach. From an assessment encompassing 25 hydroxyvitamin D level and 731 immune traits, we pinpointed three pivotal mediators: 25 hydroxyvitamin D level, CD14+ CD16- monocyte counts, and CD4+ T cell counts. Specifically:

The genus Lachnospiraceae ND3007 group exhibited an inverse relationship with 25 hydroxyvitamin D level (Beta =0.0144, 95% CI: 0.0026 to 0.0262, p=0.0169) (**Figure 5A**, **Supplementary Table 6**). In turn, elevated 25 hydroxyvitamin D level were positively associated with GCA risk (OR =0.40, 95% CI: 0.18-0.90, p=0.019) (**Figure 5B**, **Supplementary Table 6**).

The presence of genus Desulfovibrio was positively correlated with CD14+ CD16- monocyte counts (Beta = 0.1104, 95% CI: 0.0073 to 0.2136, p=0.0358). Elevated counts of these monocytes further increased the likelihood of GCA (OR = 1.23, 95% CI: 1.02-1.48, p=0.0320).

The family Bacteroidales S24-7 group showed an affirmative association with CD4+ T cell counts (Beta = 0.1324, 95% CI: 0.0213 to 0.2435, p=0.0195). This rise in CD4+ T cell counts was linked to a significant association with GCA (OR = 1.17, 95% CI: 1.04-1.31, p=0.0070).

The strength and reliability of our results were reaffirmed using methodologies consistent with our prior analyses (**Supplementary Table 6**). We found no evidence of pleiotropy or weak instrumental variables.

### Mediation proportion between gut microbiota and GCA

Utilizing MVMR analysis, we calculated the direct effect of specific gut microbiota taxa on GCA (**Table 1**). The genus Lachnospiraceae ND3007 group demonstrated a significant mediation effect through 25 hydroxyvitamin D levels, accounting for approximately 19.95% of the total effect on GCA. This was evidenced by a direct effect (BetaA) of -0.412 and a total effect (BetaC) of -0.514. Similarly, the genus Desulfovibrio revealed a notable mediation effect through CD14+ CD16- monocyte counts, contributing to 27.40% of its total influence on GCA. This was based on a direct effect (BetaA) of 0.285 and a total effect (BetaC) of 0.393. Lastly, the family Bacteroidales S24-7 group exhibited a mediation effect through CD4+ T cell counts, representing 28.51% of its total association with GCA. This was supported by a direct effect (BetaA) of 0.282 and a total effect (BetaC) of 0.395

**Table 1:** Mediation effect proportion.

Exposure	Mediator	BetaA (Direct effect)	BetaC (Total effect)	BetaA- BetaC	Mediation effect
genus Lachnospiraceae ND3007 group	25 hydroxyvitamin D level	-0.412	-0.514	-0.103	19.95%
genus Desulfovibrio	CD14+ CD16- monocyte	0.285	0.393	0.108	27.40%
family Bacteroidales S24 7group	CD4+ T cell	0.282	0.395	0.113	28.51%

## Discussion

Our MR analysis offers compelling insights into the intricate relationship between gut microbiota and GCA. The significant associations identified between specific microbial taxa and GCA underscore the potential role of gut microbiota in modulating autoimmune responses. Notably, families such as Bacteroidales S24-7group and Clostridiaceae1, along with genera like Clostridium and Desulfovibrio, emerged as key players in this relationship.

In GCA process (25), activated dendritic cells can recruit CD4 T cells, which then differentiate into Th1 and Th17 subsets, producing interferon-gamma (IFN-γ) and interleukin-17. These cytokines promote the recruitment of more immune cells, including CD4+ and CD8+ T cells and monocytes. Monocytes evolve into macrophages, which play key roles in arterial damage and inflammation. The chronic inflammation in GCA is perpetuated by immune regulatory defects and involves various pathways and cells, notably the PD-1/PD-L1 pathway and mucosal-associated invariant T cells. The central involvement of CD4 T cells and CD14+ CD16- monocytes underscores their importance in GCA’s progression and potential treatment strategies.

CD14+CD16- monocytes are classical monocytes that play a crucial role in inflammation and immune responses (26), other types of monocytes include intermediate monocytes (CD14+CD16+) and non-classical monocytes (CD14^dim^CD16+). In patients with Giant Cell Arteritis (GCA), there is a notable increase in classical monocytes, resulting in a corresponding decrease in the proportion of non-classical monocytes. This rise in classical monocytes is linked to the vascular inflammation seen in GCA, as indicated in study (27), and these monocytes can be recruited and activated by cytokines like CCL2, as reported in research (28). Migrated monocytes within the vessel wall secrete cytokines and matrix metalloproteinases, resulting in substantial vascular damage (29). Interestingly, monocytes showing a notably high per-cell expression of CCR2, which monocytes express was not affected by glucocorticoid treatment. These mean that classic monocytes play a key role on GCA. A study investigated the pathogenic effects of outer membrane vesicles (OMVs) produced by the gut bacterium, Desulfovibrio (30), which is an opportunistic pathogen prevalent in the human gut. These OMVs, with a bilayer lipid structure, contained significant proteins, including TolB, essential for toxin transport. They also triggered inflammation by releasing inflammatory mediators and induced pyroptosis in macrophages. This highlights the role of D. fairfieldensis OMVs in gut barrier compromise and inflammation. Our findings indicate that the presence of the genus Desulfovibrio is positively correlated with CD14+ CD16- monocyte counts, which further increases the likelihood of GCA.

In GCA, circulating monocytes migrate to vascular lesions and differentiate into macrophages (31), which are highly plastic cells capable of adapting to their microenvironment (32). These macrophages are broadly classified into two major subtypes: the pro-inflammatory M1 and the anti-inflammatory, tissue-repairing M2 macrophages. However, in the pathological conditions of GCA, dedicated tissue studies have revealed macrophage phenotypes displaying mixed traits of both M1 (characterized by CD64 expression) and M2 (indicated by CD206/FRβ expression) (33–35). This heterogeneity of macrophages in GCA is influenced by various soluble mediators present in the vessel wall microenvironment, including IFN-γ, PDGF, IL-17, IL-6, and GM-CSF (36). These mediators are secreted by different cell types and modulate macrophage differentiation and function. For instance, immunohistochemical analyses have identified functional diversity among tissue-infiltrating macrophages in GCA: TGF-β1(+)iNOS(−) macrophages, typically found in the adventitia near IFN(+) CD4+ T cells, contribute to the production of IL-1β and IL-6. Conversely, TGF-β1(−)iNOS(+) macrophages, located in the intimal layer of inflamed arteries, express MMP-2, a collagenase implicated in tissue destruction (37). Interestingly, the spatial distribution of different macrophage phenotypes in GCA-affected vessel walls has been noted in research (38). CD206+/YKL-40+/MMP-90+ macrophages, linked to tissue destruction, and FRβ+/CD206- macrophages, associated with intimal hyperplasia, demonstrate distinct localization patterns (39). The role of MMP-9 in this type of macrophage is particularly notable in GCA pathogenesis due to its ability to degrade elastin (40), which highlighting the complexity and context-dependence of macrophage functions in disease progression. The persistence of macrophage infiltration in the vessels of GCA patients, even during treatment (41), indicates that current therapies may not adequately suppress the local inflammatory response. Recent research has pointed to key pathways, such as the STAT6 signaling pathway (42, 43) and the influence of Jumonji domain-containing protein-3 (44), in modulating macrophage polarization. Targeting these pathways could potentially shift the balance from pro-inflammatory M1 macrophages towards anti-inflammatory M2 macrophages. Such advancements hold promise for reducing the incidence of complications like thoracic aorta and aortic dissections in GCA, offering new avenues for therapeutic intervention

CD4+ T cells are a crucial component of the adaptive immune system, playing a central role in orchestrating immune responses against a wide array of pathogens. They are often termed “helper T cells” because of their role in assisting other immune cells in their functions (45). In healthy individuals, CD4+ T cells are not typically found within arterial walls. In contrast, in GCA, there is a marked increase in CD4+ T cells within the transmural infiltrates of affected arteries (46). They can infiltrate the adventitia through vasa vasorum endothelial cells that express vascular cell adhesion molecules, such as ICAM-1 and VCAM-1 (47). They will differentiate different function T cell, which will secret a lot of cytokine, affect the vacular inflammation (48). Like Th1, Th2, Th9 and Th17 subsets (49–52), have been identified as central players in GCA’s immunopathology, orchestrating the inflammatory response in the arterial wall. High-fat diet (HFD) intake has been observed to influence the gut microbiota, notably leading to a reduction in the Bacteroidales (53). This alteration in microbiota composition is closely linked to changes in T cell populations in the small intestine lamina propria (SILP). Specifically, HFD consumption results in decreased proportions and numbers of RORγt+Th17 cells and Treg cells, which are the subset of CD4+ T cell, in the SILP. This observation aligns with findings in obese patients, who exhibit reduced Treg cells in both the small intestine and colon compared to their lean counterparts (54). The positive association of the family Bacteroidales with CD4+ T cell counts, and its subsequent link to GCA, underscores the immunomodulatory potential of gut microbiota. This is consistent with literature emphasizing the role of gut microbiota in shaping T cell responses, especially in the context of autoimmune diseases.

The Lachnospiraceae is known to produce short-chain fatty acids(SCFAs) through the fermentation of dietary fibers (55). SCFAs, especially butyrate, have anti-inflammatory properties and play a role in maintaining gut barrier integrity (56). An imbalance in this group can lead to a decrease in SCFA production, which may contribute to inflammation and autoimmune diseases (57). SCFAs play a pivotal role in modulating the immune response, particularly in the gut. For instance, they have been shown to influence the differentiation and function of colonic T cells, promoting the generation of regulatory T cells (Tregs) and limiting inflammatory Th17 cells. This balance between Tregs and Th17 is crucial for maintaining gut homeostasis (58). 25 hydroxyvitamin D is an active form of vitamin D in the body. Vitamin D has long been recognized for its role in bone health, but recent studies have highlighted its importance in immune regulation (59). It can modulate both innate and adaptive immune responses. Deficiency in vitamin D has been linked to increased susceptibility to infections and autoimmune diseases (11). In the context of the result, the negative direct effect suggests that as the presence of Lachnospiraceae decreases, there’s a decrease in 25 hydroxyvitamin D levels, which could potentially enhance inflammatory responses leading to conditions like GCA.

The comprehensive approach of our research, incorporating multiple rigorous analyses, solidifies the credibility of our outcomes. The congruence of findings across the weighted median, MR-Egger, and the primary IVW methods reinforces the strength of our deductions. Furthermore, our utilization of the MR-PRESSO strategy to detect and correct potential outliers ensures reduced biases in our results. A distinctive feature of our study is the spotlight on particular genera that showcased strong correlations with GCA. While some of these associations did not maintain statistical significance after adjustments for multiple testing, they offer valuable preliminary indications of potential biological interplays. The homogeneity in the ethnic backgrounds of our study samples, primarily of European lineage, minimizes potential biases arising from population variations.

Yet, our investigation has its set of challenges. Chief among these is the reliance on data primarily from European populations, which might introduce biases and curtails the applicability of our results to diverse ethnicities. The absence of individual-level data also limited our ability to delve into more nuanced relationships, possibly missing out on non-linear associations between microbiota, intermediaries, and GCA. Consequently, specific association patterns, like U-shaped or J-shaped curves, could have gone unnoticed.

## Future research

The compelling findings from our MR analysis pave the way for a multitude of promising research avenues. To delve deeper into the mechanisms underlying our observations, animal models, especially germ-free mice, stand out as crucial tools. By manipulating their gut microbiota composition, we can further dissect the direct influences of specific microbial taxa on immune responses and GCA progression. In the clinical realm, a detailed examination of the interplay between gut microbiota taxa and specific immune cell types and quantities in GCA patients versus controls can enhance our comprehension. Moreover, as our analysis was predominantly centered on European cohorts, replicating these findings across diverse ethnicities will not only validate our results but might also unveil population-specific microbial influences. Furthermore, functional assays can provide insights into the roles of these microbial taxa, from their metabolic outputs to their interactions with host cells. Considering the adaptability of the gut microbiota, the prospect of dietary interventions emerges as a tantalizing therapeutic strategy. Investigating the potential of specific diets to nurture protective microbial taxa could revolutionize GCA management. Integrating multi-omics data will further our holistic understanding of gut microbiota, host metabolism, and immune cross-talk in GCA. Finally, given the autoimmune nature of GCA, exploring common microbial determinants across various autoimmune disorders can unearth shared pathways and intervention points. In essence, the synergy of basic science, clinical insights, and advanced analytical tools holds immense promise for deepening our grasp on GCA and potentially reshaping its therapeutic landscape.

## Conclusion

Our comprehensive MR analysis has shed pivotal light on the intricate relationships between gut microbiota and the risk, recurrence, and long-term outcomes of GCA. By pinpointing specific microbial taxa and their associations with GCA, our research offers invaluable insights into potential microbial determinants that could influence not only the onset but also the recurrence and long-term prognosis of this autoimmune condition. These findings deepen our understanding of the multifaceted factors that contribute to GCA’s risk and its long-term recurrence patterns. Moreover, they pave the way for future investigations aimed at unraveling the complexities of GCA’s pathogenesis and its long-term implications. Our study emphasizes the significant role of gut microbiota in shaping autoimmune responses, presenting a fresh perspective on the potential determinants of GCA risk, recurrence, and sustained outcomes. Through these insights, we anticipate the emergence of innovative strategies that could profoundly influence the long-term management and prognosis of GCA.

## Data availability statement

The original contributions presented in the study are included in the article/**Supplementary Material**, further inquiries can be directed to the corresponding author/s.

## Ethics statement

The data used in this paper are publicly available, ethically approved, and the subjects have given their informed consent.

## Author contributions

MW: Conceptualization, Data curation, Formal analysis, Methodology, Resources, Software, Visualization, Writing – original draft. ZL: Conceptualization, Data curation, Investigation, Methodology, Resources, Validation, Writing – original draft. KZ: Conceptualization, Data curation, Methodology, Resources, Writing – original draft. QJ: Conceptualization, Data curation, Resources, Supervision, Validation, Writing – original draft, Writing – review & editing.
